# Effectiveness of vortioxetine in patients with major depressive disorder and comorbid Alzheimer’s disease in routine clinical practice: An analysis of a post-marketing surveillance study in South Korea

**DOI:** 10.3389/fnagi.2022.1037816

**Published:** 2023-01-09

**Authors:** Eduardo Cumbo, Michael Adair, Daniel Oudin Åstrom, Michael Cronquist Christensen

**Affiliations:** ^1^Neurodegenerative Disorders Unit, ASP 2 Caltanissetta, Caltanissetta, Italy; ^2^H. Lundbeck A/S, Valby, Denmark

**Keywords:** major depressive disorder, Alzheimer’s disease, antidepressant, vortioxetine, depressive symptoms, cognitive symptoms, cognitive performance

## Abstract

**Background:**

Vortioxetine has demonstrated procognitive effects in patients with major depressive disorder (MDD). We assessed the effectiveness and safety of vortioxetine in a cohort of patients with MDD and comorbid Alzheimer’s disease participating in a large post-marketing surveillance study in South Korea.

**Methods:**

Subgroup analysis of a 6-month, prospective, multicenter, non-interventional cohort study in outpatients with MDD with a pre-baseline diagnosis of Alzheimer’s disease receiving vortioxetine in routine care settings (*n* = 207). Patients were assessed at baseline and after 8 weeks; a subset of patients was also assessed after 24 weeks. Depression severity was assessed using the Montgomery–Åsberg Depression Rating Scale (MADRS) and Clinical Global Impression (CGI) scale, cognitive symptoms using the Perceived Deficits Questionnaire–Depression, Korean version (PDQ-K), and cognitive performance using the Digit Symbol Substitution Test (DSST).

**Results:**

Most patients were receiving a mean daily vortioxetine dose of 5 mg/day (174/190 patients; 91.6%). After 24 weeks of vortioxetine treatment, 71.4% of patients (40/56) had experienced overall clinical improvement (i.e., CGI–Improvement score ≤3) and 51.9% (28/54) had achieved remission from depressive symptoms (i.e., MADRS total score ≤10 points). Respective mean changes in MADRS, PDQ-K, and DSST total scores from baseline to week 24 were −11.5 (*p* < 0.0001), −5.1 (*p* = 0.03), and +3.8 points (*p* = 0.0524). Adverse events were reported by 27 patients (13.0%) and were mostly mild (89.2%).

**Conclusion:**

Patients with MDD and comorbid Alzheimer’s disease receiving vortioxetine in routine care settings in South Korea demonstrated clinically meaningful improvements in depressive symptoms, cognitive symptoms, and objective cognitive performance over the 6-month treatment period. Treatment with vortioxetine was well tolerated in this patient cohort, with reported adverse events consistent with the established tolerability profile of vortioxetine.

## Introduction

Among the elderly population aged ≥65 years, the global prevalence of major depressive disorder (MDD) is approximately 13% and appears to be increasing (Abdoli et al., 2022). MDD has been identified as a risk factor for the development of all types of dementia (Alzheimer’s Disease International, 2014; Cantón-Habas et al., 2020; Sáiz-Vázquez et al., 2021). A history of depression has been shown to double the risk of developing Alzheimer’s disease in later life (Ownby et al., 2006), and the greater the severity of depression, the greater the risk of subsequently developing Alzheimer’s disease (Holmquist et al., 2020). In addition, evidence suggests that late-onset depression may be a prodromal stage of Alzheimer’s disease (Enache et al., 2011; Barnes et al., 2012; Dafsari and Jessen, 2020).

Depression is also a common comorbidity in patients with Alzheimer’s disease, with up to half of all patients experiencing clinically significant depressive symptoms during the course of the disease (Starkstein et al., 2005; Enache et al., 2011). Elderly patients with MDD, particularly those with dementia, frequently do not report depressed mood and may present with less specific symptoms, such as pain, unexplained gastrointestinal symptoms, anorexia or changes in appetite, fatigue, sleep disturbances or insomnia, signs of social isolation and withdrawal, and increased dependency (Burke et al., 2019). Consequently, they may not meet the full diagnostic criteria for MDD. Depressive symptoms also tend to fluctuate over time in patients with Alzheimer’s disease, often changing over the course of the disease (Burke et al., 2019). Increasing severity of depressive symptoms has been shown to be associated with more rapid progression of Alzheimer’s disease, as assessed using the Clinical Dementia Rating (CDR) scale (Barca et al., 2017). The presence of depressive symptoms in patients with Alzheimer’s disease has also been shown to have a negative impact on patients’ ability to undertake activities of daily living and on the quality of life for both patients and their caregivers (Barca et al., 2017; Burke et al., 2019).

The relationship between depression and Alzheimer’s disease is complex and not fully understood. Available data suggest there is substantial genetic overlap between depression and Alzheimer’s disease (Monereo-Sánchez et al., 2021). Biological mechanisms that appear to underlie the association between depression and Alzheimer’s disease include dysregulation of the hypothalamic–pituitary–adrenal axis, alterations in steroid metabolism, hippocampal atrophy, inflammation, altered nerve growth factor expression, increased deposition of β-amyloid plaques, and cerebrovascular disease (Dafsari and Jessen, 2020). Neurotransmitter imbalance may also play a role. Noradrenaline, for example, has potent anti-inflammatory, neurotrophic, and neuroprotective effects, as well as influencing β-amyloid deposition, and the serotonergic system has also been shown to influence the production of β-amyloid (Dafsari and Jessen, 2020).

Selective serotonin reuptake inhibitors and serotonin–noradrenaline reuptake inhibitors are commonly used to treat depression in patients with Alzheimer’s disease (Puranen et al., 2017; Grossberg et al., 2019). However, robust evidence for the effectiveness of these agents for the treatment of depression in this population is lacking (Orgeta et al., 2017; Dudas et al., 2018; Grossberg et al., 2019; He et al., 2021). The authors of two recent systematic reviews and meta-analyses concluded that there was insufficient evidence to draw conclusions about individual antidepressant drugs (Orgeta et al., 2017; Dudas et al., 2018). A third systematic review and network meta-analysis, comprising data from 25 randomized controlled trials of 14 different antidepressants in patients with Alzheimer’s disease, found only mirtazapine and sertraline to be more effective than placebo for the treatment of symptoms of depression (He et al., 2021). In terms of cognitive function, no statistically significant differences were seen for any of the included antidepressants versus placebo. An antidepressant with broad-spectrum procognitive effects could be beneficial for treating depression in patients with Alzheimer’s disease. Some antidepressants may also have beneficial molecular effects on the pathology of Alzheimer’s disease, including effects on neurogenesis, amyloid burden, tau pathology, and inflammation (Dafsari and Jessen, 2020).

Vortioxetine is an antidepressant with a multimodal mechanism of action that is approved worldwide for the treatment of major depressive episodes in adults (Gonda et al., 2019; De Diego-Adeliño et al., 2022). The mechanism of action of vortioxetine is thought to be related to both direct modulation of serotoninergic receptor activity and inhibition of the serotonin (5-HT) transporter (Bang-Andersen et al., 2011). Vortioxetine acts as a 5-HT_3_, 5-HT_7_, and 5-HT_1D_ receptor antagonist, a 5-HT_1B_ receptor partial agonist, a 5-HT_1A_ receptor agonist, and a 5-HT transporter inhibitor, leading to modulation of neurotransmission in several systems important for mood and cognitive function—including not only the serotoninergic system, but also the noradrenaline, dopamine, histamine, acetylcholine, gamma-aminobutyric acid, and glutamate systems (Pehrson and Sanchez, 2014; Sanchez et al., 2015; Stahl, 2015). This multimodal mechanism of action is distinct from that of selective serotonin reuptake inhibitors and serotonin–noradrenaline reuptake inhibitors, and is considered to be responsible for the antidepressant and anxiolytic-like effects and improvements in cognitive function, learning, and memory observed with vortioxetine in animal studies (Mørk et al., 2013; Pehrson et al., 2013; Dale et al., 2014; du Jardin et al., 2014). In a human neuroimaging study, vortioxetine demonstrated direct beneficial effects on the neural circuitry supporting cognitive function and working memory, opposing the changes described in depression (Smith et al., 2018).

Data from randomized controlled clinical trials show that vortioxetine has broad efficacy across the spectrum of symptoms experienced by patients with MDD, including cognitive symptoms (Baldwin et al., 2016a; McIntyre et al., 2016; Thase et al., 2016; Florea et al., 2017; Christensen et al., 2018, 2021; Iovieno et al., 2021; McIntyre et al., 2021). Vortioxetine has also been shown to have significant effects on objective cognitive performance—as assessed by the Digit Symbol Substitution Test (DSST)—in patients with MDD, both in short-term randomized controlled trials (McIntyre et al., 2014, 2016, 2017; Mahableshwarkar et al., 2015; Baune et al., 2018) and open-label studies (Chokka et al., 2019; Fagiolini et al., 2021; Mattingly et al., 2022). Treatment with vortioxetine is associated with improvements in multiple domains of cognitive functioning in patients with MDD, including global cognition, executive functioning, processing speed, and attention (Blumberg et al., 2020), as well as working memory as assessed using the Rey Auditory Verbal Learning Test (McIntyre et al., 2014).

Vortioxetine has also been shown to have beneficial effects on mood and cognitive performance, including working memory, in older adults with depression (Katona et al., 2012; Bishop et al., 2021), including those with mild Alzheimer’s disease and depressive symptoms (Cumbo et al., 2019). Vortioxetine treatment was associated with significant improvements in cognitive function in an open-label study in community-dwelling older adults with mild cognitive impairment (Tan and Tan, 2021). A small retrospective cohort study has also demonstrated improvements in behavioral and psychological symptoms of dementia during treatment with vortioxetine (Valverde et al., 2020).

The aim of the present analysis was to assess the effectiveness and tolerability of vortioxetine in a cohort of patients with MDD and comorbid Alzheimer’s disease who were participating in a large post-marketing surveillance study in South Korea (Kim et al., under review^1^).

## Materials and methods

### Study design and participants

This was a subgroup analysis of a mandatory 6-month, prospective, multicenter, non-interventional post-marketing surveillance study conducted under conditions of routine clinical practice in 72 hospitals and clinics in South Korea from 13 June 2016 to 19 May 2020. Patients were receiving vortioxetine for the treatment of MDD at their physician’s discretion according to the local approved label, either as first-line treatment for the current depressive episode or switching from another antidepressant due to inadequate response or lack of tolerability. In accordance with local prescribing information, the starting dose of vortioxetine was 10 mg/day in patients aged 19–64 years and 5 mg/day in those aged ≥65 years. Vortioxetine dosage could be adjusted within the approved dose range of 5–20 mg/day at the treating physician’s discretion based on treatment response. Patients with a history of monoamine oxidase inhibitor use within 14 days before enrollment were excluded. Use of other medications was permitted at the investigator’s discretion. The current analysis included only patients with a recorded diagnosis of Alzheimer’s disease at baseline.

The study was conducted in accordance with the regulations of the Ministry of Food and Drug Safety (MFDS) in South Korea and was approved by a central institutional review board or institutional bioethics committee designated by the Ministry of Health and Welfare. Written informed consent was provided by all patients or their legal representative prior to study participation.

### Study assessments

Data were collected at routine clinic visits at baseline and after 8 and 24 (±2) weeks of vortioxetine treatment. Of note, the study visit at 8 weeks was planned for all patients; however, in accordance with MFDS guidelines, only 10% of patients participating in the post-marketing surveillance study were required to attend the long-term follow-up visit at 24 weeks. Concomitant drug usage was recorded at baseline and during the study.

Severity of depressive symptoms was assessed by physicians using the Montgomery–Åsberg Depression Rating Scale (MADRS) and the Clinical Global Impression (CGI) scale. The MADRS is a well-established scale for assessing the severity of depressive symptoms, and is designed to be sensitive to the effects of antidepressant treatment in patients with MDD (Montgomery and Åsberg, 1979). Each of the 10 items is scored on a 7-point scale, ranging from 0 (absent) to 6 (severe). MADRS total score ranges from 0 to 60 points, with higher scores indicating greater depressive symptom severity. The CGI–Severity (CGI-S) scale was used at baseline to provide a measure of overall disease severity over the past 7 days. CGI-S score ranges from 1 (normal, not at all ill) to 7 (extremely ill; Guy, 1976; Busner and Targum, 2007). Data on the CGI-S were not collected at subsequent visits. Change in overall disease severity at weeks 8 and 24 was assessed using the CGI–Improvement (CGI-I) scale. CGI-I score ranges from 1 (very much improved) to 7 (very much worse; Guy, 1976; Busner and Targum, 2007).

Cognitive symptoms were assessed using the Korean version of the patient-reported Perceived Deficits Questionnaire–Depression (PDQ-K; Kim et al., 2016). This 20-item scale assesses the severity of self-reported cognitive symptoms over the previous 7 days across four domains of cognitive function: attention/concentration, retrospective memory, prospective memory, and organization/planning. Respondents rate the frequency of each item on a 5-point Likert scale ranging from 0 (never) to 4 (almost always). PDQ-K total score ranges from 0 to 80 points, with a higher score indicating more severe cognitive symptoms.

Cognitive performance was assessed using the DSST (Wechsler, 1981). This ‘pencil-and-paper’ neuropsychological coding test involves substituting simple symbols for numbers. The DSST score is the number of correct number/symbol matches achieved during a 90-s period. The total score ranges from 0 to 133, with higher scores indicating better cognitive performance.

Adverse events (AEs) spontaneously reported to or observed by the investigator were recorded according to local regulations. AEs were graded by severity (mild, moderate, and severe) and system-organ class based on World Health Organization Adverse Reactions Terminology.

### Statistical analysis

Safety was assessed in all eligible patients who initiated treatment with vortioxetine (safety analysis set). For each endpoint, effectiveness was assessed in all eligible patients who initiated treatment with vortioxetine and who had a valid baseline assessment and at least one post-baseline assessment (effectiveness analysis set).

Data are summarized descriptively. Summary statistics (mean and standard deviation [SD]) are presented for continuous variables, and counts and percentages are presented for categoric or binary variables. Analyses were based on observed cases; missing data were not imputed.

Change in outcome assessment scores from baseline at each visit was assessed by paired *t*-test. The proportion of patients achieving improvement and response was calculated based on the CGI-I score at weeks 8 and 24. Improvement was defined as a CGI-I score of ≤3 and response as a CGI-I score of ≤2. Rates of MADRS response and remission were also assessed, with response defined as a ≥50% reduction in MADRS total score from baseline and remission defined as a MADRS score of ≤10.

Exploratory analyses were also performed for all effectiveness endpoints stratified according to whether or not patients had received prior antidepressant therapy.

All analyses were conducted using R statistical software version 4.2.0 (R Core Team, 2022). Significance was set at *p* < 0.05.

## Results

### Patient population

A total of 207 patients with MDD and comorbid Alzheimer’s disease were enrolled and received treatment with vortioxetine (safety analysis set). The effectiveness analysis set comprised 139 patients at baseline, 135 patients at week 8, and 59 patients at week 24. Patient demographics and clinical characteristics at baseline are shown in Table 1. Patients were predominantly female (72%). Mean (SD) age was 77.7 (6.1) years and most patients (92.2%) were aged ≥70 years. The most commonly reported comorbid medical conditions were hypertension (21.3% of patients), diabetes mellitus (9.2%), and gastroesophageal reflux disease (7.2%).

**Table 1:** Patient demographic and clinical characteristics at baseline (safety analysis set).

Characteristic	Safety population (*N* = 207)
Sex, *n* (%)	
Female	149 (72.0)
Male	58 (28.0)
Age (years), mean ± SD	77.7 ± 6.1
>70 years, *n* (%)	193 (93.2)
Weight (kg), mean ± SD^a^	57.6 ± 10.5
Comorbid medical conditions in ≥5% of patients, *n* (%)	
Hypertension	44 (21.3)
Diabetes mellitus	19 (9.2)
Gastroesophageal reflux disease	15 (7.2)
Hyperlipidemia	13 (6.3)
Type 2 diabetes mellitus	13 (6.3)
Other cognitive disorder	13 (6.3)
Benign prostatic hyperplasia	12 (5.8)
Cataract	11 (5.3)
Duration of MDD (days), mean ± SD^b^	545.0 ± 1449.7
Baseline assessment scores, mean ± SD	
CGI-S	3.8 ± 0.8
MADRS^c^	25.9 ± 8.9
PDQ-K^d^	37.7 ± 17.1
DSST^e^	13.0 ± 9.2
Vortioxetine as first-line treatment for current MDE, *n* (%)	125 (60.4)
Vortioxetine dosage, *n* (%)^f^	
5 mg/day	174 (91.6)
10 mg/day	12 (6.3)
15 mg/day	4 (2.1)
20 mg/day	0

CGI-S, Clinical Global Impression–Severity (score range 1–7); DSST, Digit Symbol Substitution Test (score range 0–133); MADRS, Montgomery–Åsberg Depression Rating Scale (score range 0–60); MDD, major depressive disorder; MDE, major depressive episode; PDQ-K, Perceived Deficits Questionnaire–Depression, Korean version (score range 0–80); SD, standard deviation. ^a^*n* = 143; ^b^*n* = 188; ^c^*n* = 139; ^d^*n* = 111; ^e^*n* = 43; ^f^*n* = 190.

The mean (SD) duration of MDD was 545.0 (1449.7) days. Patients had moderately severe depressive and cognitive symptoms at baseline. Mean (SD) CGI-S and MADRS total scores at baseline were 3.8 (0.8) and 25.9 (8.9) points, respectively, and the mean (SD) PDQ-K total score was 37.7 (17.1) points. As might be expected in a population of patients with comorbid Alzheimer’s disease, cognitive performance was very impaired at baseline, with a mean (SD) baseline DSST total score of 13.0 (9.2) points (possible maximum, 133 points).

Almost two-thirds of patients (60.4%) were receiving vortioxetine as first-line treatment for the current depressive episode. Of the 82 patients switching to vortioxetine from another antidepressant, most (98.8%) were switching due to lack of effectiveness of their prior therapy; one patient was switching due to lack of tolerability. Data on previous antidepressant treatment were available for 32 patients. In these patients, the most commonly prescribed antidepressants were escitalopram (*n* = 18), trazodone (*n* = 6), paroxetine (*n* = 5), and mirtazapine (*n* = 4); some patients were receiving more than one antidepressant.

Of the 190 patients with available vortioxetine dose data, most (91.6%) were receiving a mean daily dosage of 5 mg/day, 6.3% were receiving 10 mg/day, and 2.1% were receiving 15 mg/day (Table 1).

Most patients (98.1%) were receiving concomitant medication at baseline (most commonly, drugs for the treatment of Alzheimer’s disease). A total of 177/207 (85.5%) patients were receiving an acetylcholinesterase inhibitor and/or memantine. An additional 10 patients (4.8%) were receiving choline alfoscerate, an acetylcholine precursor, not in combination with any of the above treatments. The most common treatment for Alzheimer’s disease was monotherapy with the acetylcholinesterase inhibitor donepezil (received by 149/207 patients [72.0%]).

With regard to other psychotropic medications used at baseline, 13 patients were receiving antipsychotics, 11 were receiving anxiolytics, and four were receiving hypnotics. The most commonly used non-psychiatric concomitant medications were drugs for acid-related disorders (antacids or drugs for peptic ulcer disease and gastroesophageal reflux disease; *n* = 58), drugs for diabetes (insulins and blood-glucose-lowering drugs; *n* = 40), and drugs for functional gastrointestinal disorders (*n* = 39).

### Effectiveness

**Figure 1:**
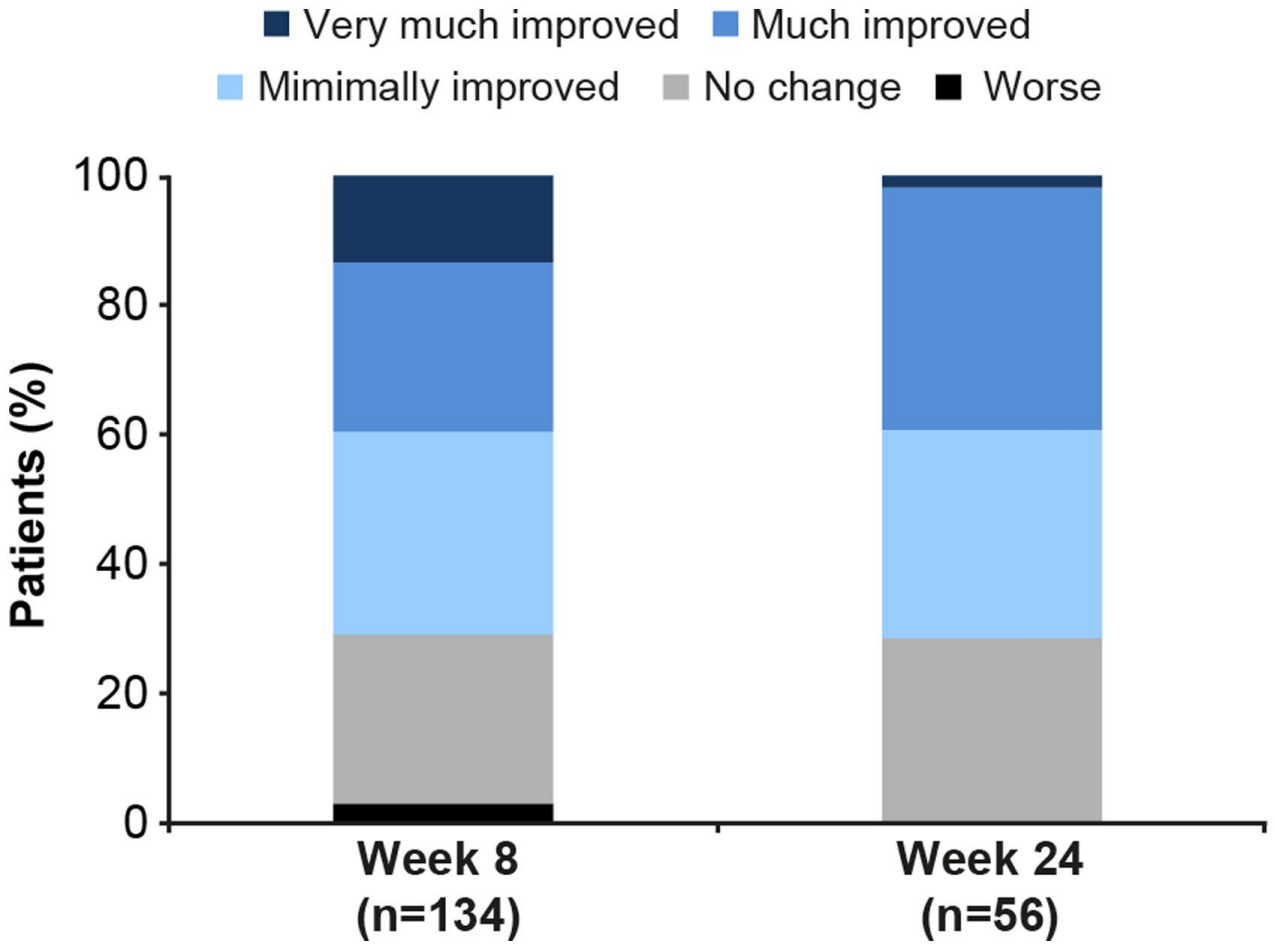
Clinical Global Impression–Improvement (CGI-I) scores in patients with major depressive disorder and comorbid Alzheimer’s disease after 8 and 24 weeks of vortioxetine treatment (effectiveness analysis set). Very much improved, CGI-I score = 1; Much improved, CGI-I score = 2; Minimally improved, CGI-I score = 3; No change, CGI-I score = 4; Worse, CGI-I score ≥5.

Based on CGI-I score, most patients experienced clinically meaningful improvement in overall MDD severity over the 6 months of vortioxetine treatment (Figure 1). Overall improvement (i.e., a CGI-I score ≤3) was seen in 95/134 patients (70.9%) at week 8 and 40/56 (71.4%) at week 24. Response (i.e., a CGI-I score ≤2) was achieved by 53/134 patients (39.6%) at week 8 and 22/56 patients (39.3%) at week 24. The median CGI-I score was 3 (interquartile range, 2) at both time points.

**Figure 2:**
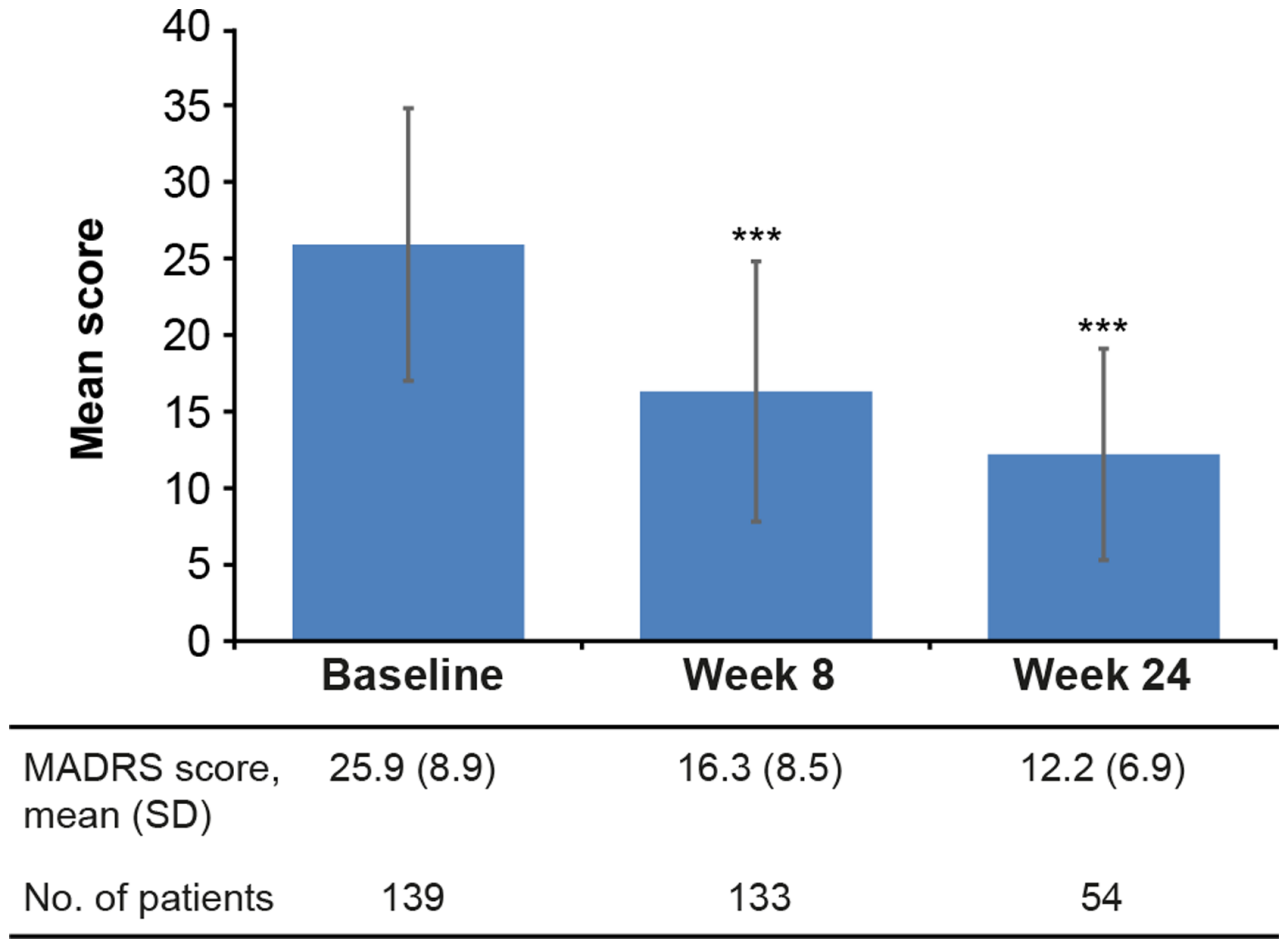
Mean (SD) MADRS total score at baseline and after 8 and 24 weeks of vortioxetine treatment (effectiveness analysis set). ***p < 0.0001 vs. baseline. MADRS, Montgomery–Åsberg Depression Rating Scale (score range 0–60); SD, standard deviation.

**Figure 3:**
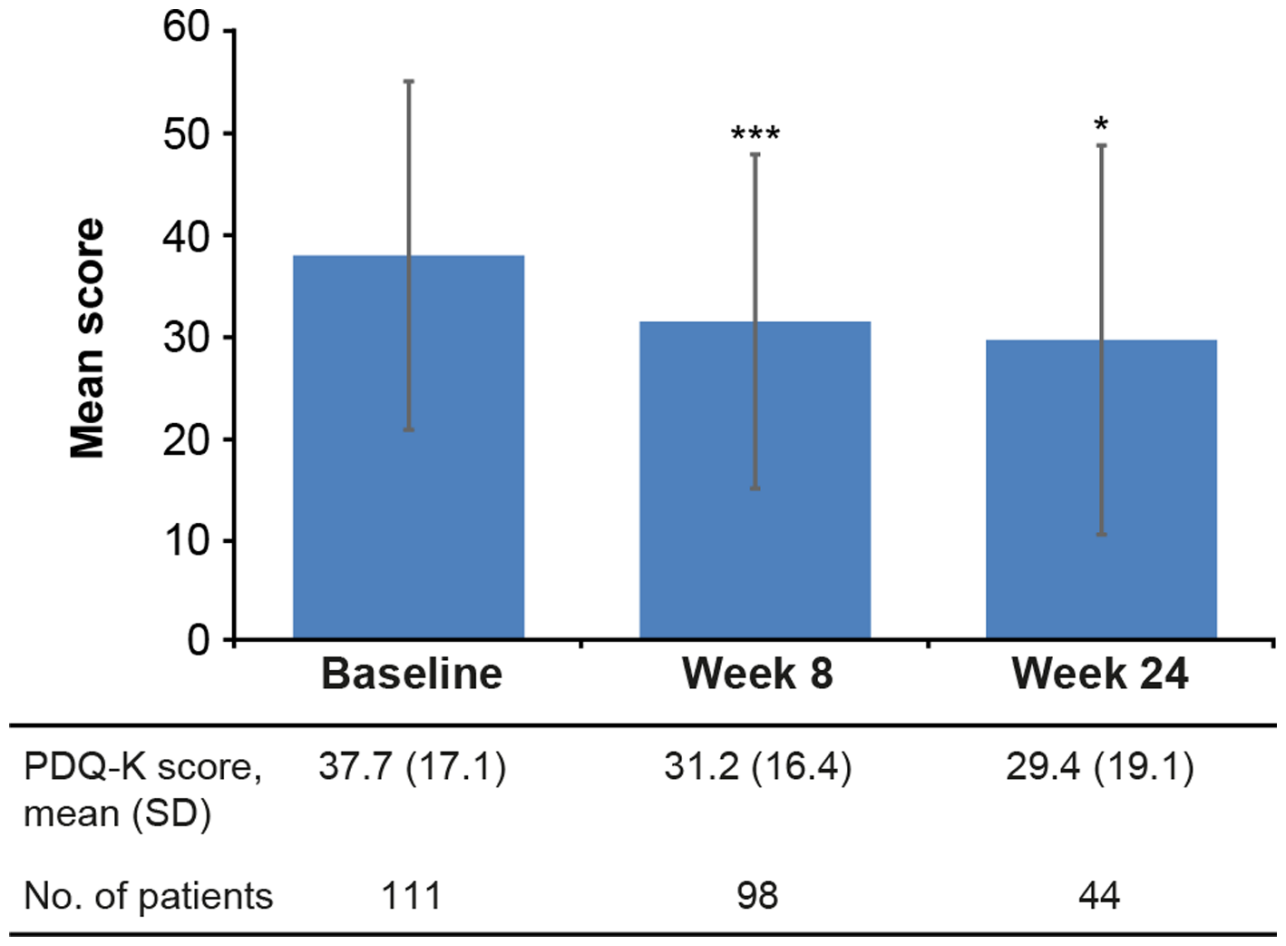
Mean (SD) PDQ-K total score at baseline and after 8 and 24 weeks of vortioxetine treatment (effectiveness analysis set). *p < 0.05 and ***p < 0.0001 vs. baseline. PDQ-K, Perceived Deficits Questionnaire–Depression, Korean version; SD, standard deviation.

Statistically significant reductions in the severity of depressive (Figure 2) and cognitive symptoms (Figure 3) were observed over the 6 months of vortioxetine treatment. Mean (SD) change in MADRS total score from baseline was −9.6 (10.3) points at week 8 and −11.5 (8.8) points at week 24 (both *p* < 0.0001 vs. baseline; Table 2). For depressive symptoms, response (i.e., ≥50% reduction in MADRS total score from baseline) was seen in 48/133 patients (36.1%) at week 8 and 27/54 patients (50.0%) at week 24. Remission from depressive symptoms (i.e., MADRS total score ≤10) was achieved by 36/133 patients (27.1%) at week 8 and 28/54 (51.9%) at week 24. Mean (SD) change in PDQ-K total score from baseline was −6.4 (13.1) points at week 8 (*p* < 0.0001 vs. baseline) and −5.1 (15.0) points at week 24 (*p* = 0.03 vs. baseline; Table 2).

**Table 2:** Change in MADRS, PDQ-K, and DSST scores from baseline after 8 and 24 weeks of vortioxetine treatment (effectiveness analysis set).

Outcome^a^	Week 8	Week 24
MADRS		
Mean (SD)	−9.6 (10.3)	−11.5 (8.8)
*p* value	<0.0001	<0.0001
*n*	133	54
PDQ-K		
Mean (SD)	−6.4 (13.1)	−5.1 (15.0)
*p* value	<0.0001	0.03
*n*	98	43
DSST		
Mean (SD)	1.7 (7.2)	3.8 (8.1)
*p* value	0.1653	0.0524
*n*	36	20

DSST, Digit Symbol Substitution Test (score range 0–133); MADRS, Montgomery–Åsberg Depression Rating Scale (score range 0–60); *n*, number of patients with available data; PDQ-K, Perceived Deficits Questionnaire–Depression, Korean version (score range 0–80); SD, standard deviation. ^a^For all values, mean (SD) change from baseline is shown and *p* values are for paired *t*-test versus baseline.

**Figure 4:**
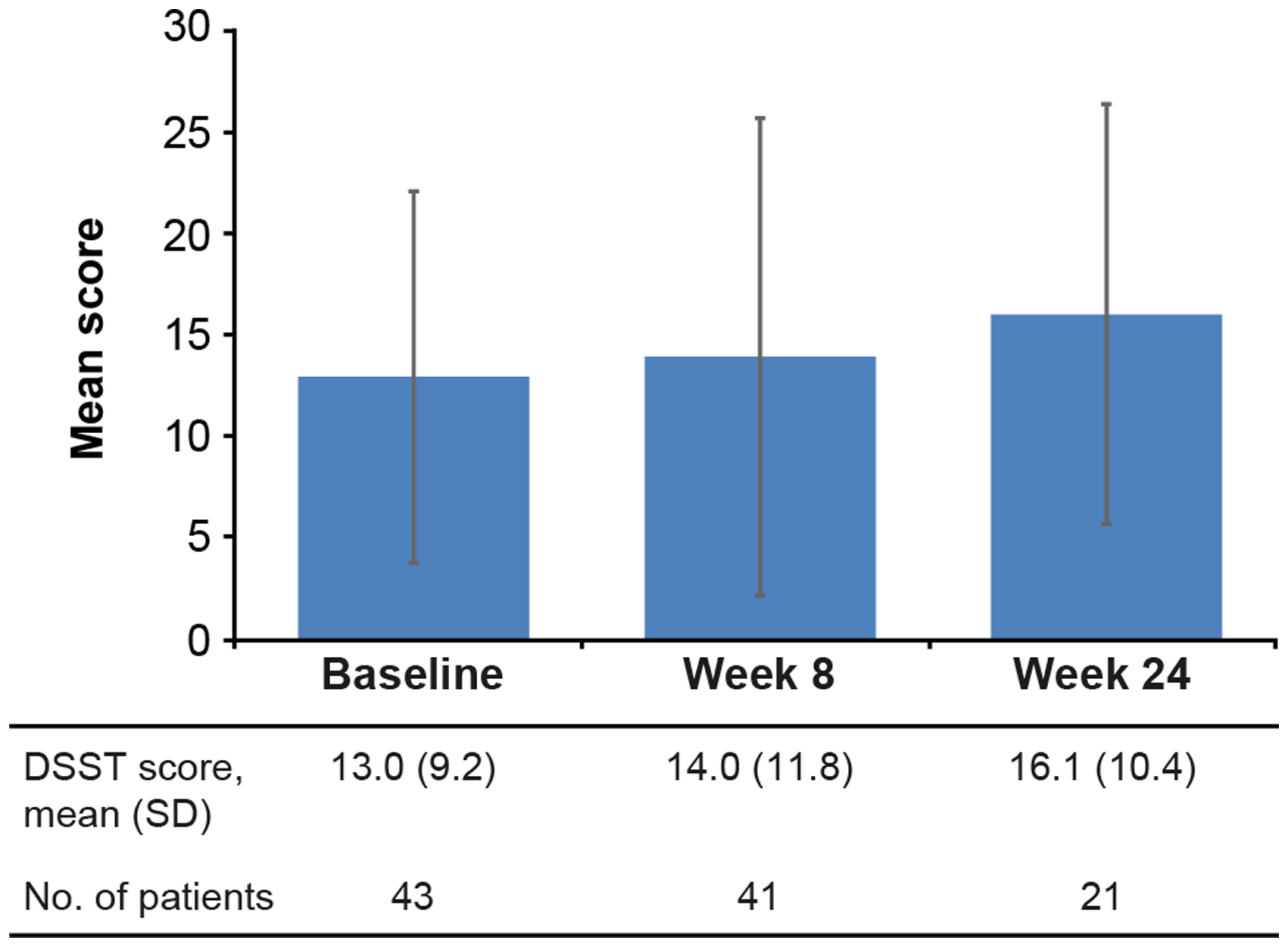
Mean (SD) DSST total score at baseline and after 8 and 24 weeks of vortioxetine treatment (effectiveness analysis set). DSST, Digit Symbol Substitution Test; SD, standard deviation.

Improvement in objective cognitive performance was also observed (Figure 4). Mean (SD) change in DSST total score from baseline was +1.7 (7.2) points at week 8 and +3.8 (8.1) points at week 24 (difference vs. baseline, not significant at either time point; Table 2).

Results of the exploratory analyses of effectiveness endpoints stratified according to whether or not patients had received prior antidepressant therapy are summarized in Supplementary Table S1. Mean MADRS and DSST scores at baseline were similar in the two patient groups; however, mean PDQ-K score at baseline was significantly higher (i.e., worse) in patients who had received prior antidepressant therapy (41.8 vs. 34.6 in treatment-naïve patients, *p* = 0.03). No statistically significant differences were observed between the two patient groups at either week 8 or 24, with the exception at week 8 of a significantly greater proportion of treatment-naïve patients achieving remission (i.e., MADRS total score ≤10) than those previously treated with antidepressants (36.6% vs. 11.8%; *p* = 0.003).

### Safety

A total of 37 AEs were recorded in 27 patients (13.0%) over the 24 weeks of vortioxetine treatment (Table 3). No unexpected AEs were reported; the only AEs reported by more than one patient were decreased appetite (four patients, 1.9%) and nausea, dizziness, and lower limb fracture (each reported by two patients, 1.0%). AEs were mostly mild in intensity (89.2%); only four moderate and no severe AEs were recorded. No significant change in mean body weight from baseline was observed over the 24 weeks of vortioxetine treatment (mean [SD] change in body weight from baseline, −0.5 [3.4] kg at week 24).

**Table 3:** Summary of AEs (safety analysis set; *N* = 207).

	No. of patients, *n* (%)	No. of events
Any AE	27 (13.0)	37
Serious AEs	9 (4.3)	12
AEs occurring in ≥2 patients		
Decreased appetite	4 (1.9)	4
Nausea	2 (1.0)	2
Dizziness	2 (1.0)	2
Lower limb fracture	2 (1.0)	2

AE, adverse event.

## Discussion

This subgroup analysis was undertaken to evaluate the effectiveness and tolerability of vortioxetine in patients with MDD and comorbid Alzheimer’s disease. Overall data are limited concerning the effectiveness of antidepressants in this specific patient population. Our findings demonstrate vortioxetine to be effective and well tolerated in patients with MDD and comorbid Alzheimer’s disease receiving treatment in routine care settings in South Korea. Significant and sustained reductions in the severity of both core depressive symptoms (assessed using the MADRS) and cognitive symptoms (assessed using the PDQ-K) were observed over 6 months of follow-up. Notably, just over half of all patients achieved remission from their depressive symptoms after 6 months of vortioxetine treatment. The majority of patients also demonstrated overall clinical improvement based on the CGI-I score, with a CGI-I score of ≤3 in approximately 70% of patients at weeks 8 and 24. Approximately 40% of patients demonstrated a response on the CGI-I scale (i.e., score ≤ 2) at both time points.

It should be noted that the majority of patients included in this analysis were receiving vortioxetine 5 mg/day, which is the lower end of the approved dose range. Vortioxetine has been shown to have dose-dependent efficacy in patients with MDD, with the greatest therapeutic effects achieved at the maximum dosage of 20 mg/day (Baldwin et al., 2016a; McIntyre et al., 2016; Thase et al., 2016; Florea et al., 2017; Christensen et al., 2018, 2021; Iovieno et al., 2021; McIntyre et al., 2021). Nevertheless, the observed improvement in depressive symptom severity, as assessed by change in mean MADRS total score from baseline at weeks 8 and 24 (approximately 10 and 12 points, respectively), was greater than the threshold that has been suggested to be clinically meaningful in adults with MDD alone (i.e., a 6–10 point reduction in MADRS total score; Duru and Fantino, 2008; Hudgens et al., 2021; Turkoz et al., 2021). The observed reduction in MADRS total score was also greater than that reported after 8 weeks of vortioxetine treatment in an earlier randomized controlled study in elderly patients with MDD alone (~5 points; Katona et al., 2012). In that study, reported rates of MADRS response and remission after 8 weeks of vortioxetine treatment were 60% and 34%, respectively (Katona et al., 2012); in the present study, approximately one-third of patients had achieved a response and just over one-quarter had achieved remission of depressive symptoms after 8 weeks of vortioxetine treatment.

In the present analysis, cognitive performance was assessed using the DSST (Wechsler, 1981). The DSST is widely used in trials of antidepressant therapy as it covers aspects of cognitive functioning known to be impaired in patients with MDD, including processing speed, executive functioning, and attention (Jaeger, 2018). Although patients did not undergo any dementia-specific neuropsychological evaluations, the multifactorial properties of the DSST allow for this test to be sensitive to different types of cognitive impairment (e.g., age-related decline vs. brain disease or damage; Jaeger, 2018). The DSST also shows high test–retest reliability (Jaeger, 2018).

Of note, objective cognitive performance was maintained over the 6 months of vortioxetine treatment. The observed improvements in DSST score seen in our study appear clinically meaningful in a community setting, with an increase in the mean DSST score of approximately 4 points observed over the 24 weeks of vortioxetine treatment. While this change from baseline did not reach statistical significance, it is in line with the results of previous studies in more homogeneous patient groups (Katona et al., 2012; McIntyre et al., 2014; Mahableshwarkar et al., 2015; Mattingly et al., 2022). In a randomized controlled trial in elderly patients with MDD without a diagnosis of dementia, mean change in DSST score after 8 weeks of vortioxetine treatment was approximately 3 points (Katona et al., 2012); this is consistent with the mean change from baseline of approximately 2 points after 8 weeks of vortioxetine treatment in the present study. Statistically significant improvements in the severity of patient-reported cognitive symptoms, as assessed by the PDQ-K, were also observed over 6 months of vortioxetine treatment, supporting the DSST findings.

These DSST findings suggesting maintenance of cognitive performance (integrated cognitive functioning, including executive function, processing speed, attention, spatial perception, and visual scanning) in patients with MDD and comorbid Alzheimer’s disease over the 24 weeks of vortioxetine treatment should be interpreted with caution, as this assessment was not performed in all patients. However, the observed improvements in cognitive symptoms and performance in this analysis are noteworthy given the generally progressive nature of cognitive impairment in patients with Alzheimer’s disease (Scheltens et al., 2021).

The safety data collected in this analysis further support the established favorable tolerability profile of vortioxetine in patients with MDD (Baldwin et al., 2016b). In line with previous findings in elderly patients without the presence of any significant neurodegenerative disorder (Katona et al., 2012), vortioxetine was well tolerated in this cohort of elderly patients with comorbid Alzheimer’s disease. As in other observational studies (Chin et al., 2018; Yang et al., 2021; Mattingly et al., 2022; Kim et al., under review), AEs were mostly mild to moderate in intensity and were consistent with the known tolerability profile of vortioxetine. In addition, no changes in body weight were seen over the 6 months of vortioxetine treatment.

Clinical improvements have also been observed in a 12-month, open-label, observational study of vortioxetine in patients with mild Alzheimer’s disease and depressive symptoms in Italy (Cumbo et al., 2019). A total of 108 patients were included in that study, of whom 36 received vortioxetine 15 mg/day and 72 received other drugs for depression (escitalopram, paroxetine, venlafaxine, sertraline, or bupropion). Statistically significant improvements in both mood symptoms (assessed using the Hamilton Depression Scale and Cornell Scale for Depression in Dementia) and cognitive function (assessed using the Mini-Mental State Examination [MMSE] and other neuropsychological tests) were seen over 12 months of vortioxetine treatment. At 12 months, the mean improvement in MMSE total score was 2.9 points in the vortioxetine group (*p* < 0.001 vs. baseline) compared with 0.4 points in the control group (not significant vs. baseline). The difference between the vortioxetine and control groups was statistically significant (2.5 points; *p* = 0.05). Improvements in secondary measures of cognitive performance were also seen in the vortioxetine group, with significant improvements from baseline and versus the control group seen for Attentive Matrices and Raven Colored Progressive Matrices scores. Vortioxetine was found to be well tolerated, with nausea and headache being the most frequently reported AEs (each reported by 9% of patients over the 12-month study period).

In another study, elderly adults (≥65 years) with age-related cognitive decline who received vortioxetine 10 mg/day in combination with computerized cognitive training experienced significantly greater improvements in global cognitive performance, as assessed by the NIH Toolbox Cognition Battery fluid cognition composite score, than those who received placebo over a period of 6 months (Lenze et al., 2020). Treatment with vortioxetine was generally well tolerated, with nausea being the most commonly reported AE.

Similarly, in an open-label study, community-dwelling older adults with mild cognitive impairment demonstrated significant and sustained improvements in cognitive function, as assessed using the Montreal Cognitive Assessment and DSST, during treatment with vortioxetine 5–10 mg/day, with a mean change in DSST total score from baseline to 6 months of approximately 12 points (Tan and Tan, 2021). Almost 90% of patients demonstrated overall improvement in cognitive impairment, as assessed by the CDR scale. A significant improvement in CDR global score was seen over the 6-month treatment period, mainly due to improvement in the CDR memory score. In fact, the mean CDR global score after 6 months of vortioxetine was 0.13 points, indicative of normal cognitive function. Treatment with vortioxetine was well tolerated. Nausea was the most common adverse drug reaction, and was reported by only two patients (1.8%).

In contrast, in another recent short-term, randomized, double-blind, placebo-controlled study of vortioxetine in patients with Alzheimer’s disease experiencing severe depression, no statistically significant differences between groups were seen in depressive symptoms, cognitive function (assessed using a range of neuropsychological tests), or overall patient functioning after 12 weeks of treatment (Jeong et al., 2022). However, it should be noted that participants in this study had more severe dementia, as assessed by mean MMSE scores, than those in the recent observational study in Italy that demonstrated the effectiveness of vortioxetine in patients with mild Alzheimer’s disease (14 vs. 21 points, respectively) (Cumbo et al., 2019).

The main strengths of the current analysis are that it was conducted in a large cohort of patients with MDD and comorbid Alzheimer’s disease receiving treatment in routine practice settings. A further strength is the longitudinal 24-week study design, which offers significant advantages over cross-sectional and prospective studies of shorter follow-up in terms of evaluating the effect of treatment on symptoms of depression and functional and cognitive status in patients with Alzheimer’s disease.

A key limitation is the fact that this was a subgroup analysis of a larger study, and was therefore not specifically designed to assess the effectiveness and tolerability of vortioxetine in patients with comorbid Alzheimer’s disease. As such, data on the severity of baseline cognitive impairment in the study population were limited and patients were not assessed using scales commonly applied to assess disease severity in patients with Alzheimer’s disease, such as the MMSE. While the DSST was used to assess objective cognitive performance, this test was not completed at both baseline and subsequent visits in the majority of patients. It is possible that this is because the DSST was only administered to less severely cognitively impaired individuals; if so, this could be a potential source of bias. Furthermore, as this subgroup analysis is based on observational data obtained in real-world clinical practice, there is no untreated control group that may have facilitated assessment of the clinical relevance of the improvements in cognitive performance observed during vortioxetine treatment.

Other possible limitations include the lack of data concerning previous antidepressant therapy for all patients switching from other drugs, and the lower number of patients included in the effectiveness analysis set at week 24 than at week 8 (most likely due to the fact that the long-term follow-up visit was not required for all patients). In accordance with MFDS guidelines, only 10% of patients participating in the post-marketing surveillance study were required to attend the week 24 assessment. This option was not defined at the patient level; rather, the decision to assess patients at week 24 was agreed with the investigator at specific study sites prior to any patients entering the study at that site. At these sites, all patients who remained on vortioxetine at 24 weeks were assessed. In this analysis, data were available for 59 patients at week 24 (i.e., 28.5% of included patients).

## Conclusion

In summary, results of this analysis demonstrate the real-world effectiveness and tolerability of vortioxetine for the treatment of MDD in patients with Alzheimer’s disease. Clinically meaningful improvements in depressive symptoms, cognitive symptoms, and objective cognitive performance were observed over the 6 months of vortioxetine treatment. Vortioxetine was well tolerated in this patient cohort, most of whom were receiving concomitant medication. Additional prospective, controlled, long-term studies are required to further explore the potential cognitive benefits of vortioxetine in patients with MDD and Alzheimer’s disease.

## Data availability statement

The data supporting the findings of this analysis are available within the manuscript/Supplementary material. H. Lundbeck A/S may be contacted for further data sharing.

## Ethics statement

The study was conducted in accordance with Korean Ministry of Food and Drug Safety regulations and was approved by a central institutional review board or institutional bioethics committee designated by the Ministry of Health and Welfare. Informed consent for participation was provided for all patients.

## Author contributions

MA was responsible for the conception of this analysis. DOÅ was responsible for data analysis. The authors are entirely responsible for the scientific content of this paper. All authors contributed to critical interpretation of the data, drafting the manuscript and manuscript revision, and read and approved the final version.

## Funding

This analysis was funded by H. Lundbeck A/S, whose personnel contributed to the data analysis, review of the data, and review of the manuscript. Medical writing assistance was provided by Jennifer Coward of Piper Medical Communications, funded by H. Lundbeck A/S.

## Conflict of interest

EC has participated in advisory boards and pharmaceutical industry-sponsored symposia for Pfizer, GSK, Eli Lilly, UCB Pharma, Lundbeck, Novartis Pharma, and Neopharmed Gentili. MA, DOÅ, and MCC are employees of H. Lundbeck A/S.

The handling editor AF declared a past co-authorship with the author MCC.

## Publisher’s note

All claims expressed in this article are solely those of the authors and do not necessarily represent those of their affiliated organizations, or those of the publisher, the editors and the reviewers. Any product that may be evaluated in this article, or claim that may be made by its manufacturer, is not guaranteed or endorsed by the publisher.

## Footnotes

^1^Kim, D. H., Moon, S. W., Kim, J. W., Lee, K. Y., Reines, E. H., Lee, M., et al. (Under review). Safety and effectiveness of vortioxetine for major depressive disorder: Real-world evidence from a population-based study in South Korea.
