# Supplementary material for: Effectiveness of vortioxetine in patients with major depressive disorder and comorbid Alzheimer’s disease in routine clinical practice: An analysis of a post-marketing surveillance study in South Korea
Source: Front Aging Neurosci. 2023 Jan 9;14:1037816. doi: 10.3389/fnagi.2022.1037816 (PMC9868833; doi:10.3389/fnagi.2022.1037816)
Supplement: Supplementary file 1 [file Table_1.DOCX]

**Effectiveness of Vortioxetine in Patients with Major Depressive Disorder and Comorbid Alzheimer’s Disease in Routine Clinical Practice: An Analysis of a Post-Marketing Surveillance Study in South Korea**

**Supplementary Table 1.** Exploratory analysis of vortioxetine effectiveness according to prior antidepressant therapy (effectiveness analysis set)

| Outcome | Treatment-naïve  (n=84) | Prior therapy  (n=55) | *p* value |
| --- | --- | --- | --- |
| Baseline scores, mean ± SD |  |  |  |
| MADRS | 25.4 ± 9.2 | 26.6 ± 8.5 | 0.462 |
| PDQ-K | 34.6 ± 17.6 | 41.8 ± 15.7 | **0.026** |
| DSST | 13.8 ± 9.6 | 11.5 ± 8.5 | 0.459 |
| Week 8 |  |  |  |
| Mean change from baseline,  mean ± SD |  |  |  |
| MADRS | −10.7 ± 10.5 | −7.8 ± 9.8 | 0.125 |
| PDQ-K | −5.5 ± 13.9 | −7.8 ± 12.0 | 0.402 |
| DSST | 2.3 ± 8.0 | 0.4 ± 5.3 | 0.458 |
| CGI-I≤3 (i.e., improvement), n (%) | 61 (74.4) | 34 (65.4) | 0.356 |
| CGI-I≤2 (i.e., response), n (%) | 35 (42.7) | 18 (34.6) | 0.454 |
| MADRS response,^a^ n (%) | 35 (42.7) | 13 (25.5) | 0.068 |
| MADRS remission,^b^ n (%) | 30 (36.6) | 6 (11.8) | **0.003** |
| Week 12 |  |  |  |
| Mean change from baseline,  mean ± SD |  |  |  |
| MADRS | −12.5 ± 9.9 | −10.3 ± 7.0 | 0.368 |
| PDQ-K | −5.6 ± 16.7 | −4.6 ± 13.2 | 0.829 |
| DSST | 4.5 ± 9.2 | 2.9 ±7.0 | 0.679 |
| CGI-I≤3 (i.e., improvement), n (%) | 26 (81.2) | 14 (58.3) | 0.114 |
| CGI-I≤2 (i.e, response), n (%) | 15 (46.9) | 7 (29.2) | 0.286 |
| MADRS response,^a^ n (%) | 18 (58.1) | 9 (39.1) | 0.271 |
| MADRS remission,^b^ n (%) | 20 (64.5) | 8 (34.8) | 0.059 |

CGI-I, Clinical Global Impression–Improvement (score range 1–7); Clinical Global Impression–Severity (score range 1–7); DSST, Digit Symbol Substitution Test (score range 0–133); MADRS, Montgomery–Åsberg Depression Rating Scale (score range 0–60); PDQ-K, 20-item Perceived Deficits Questionnaire–Depression Korean Version (score range 0–80)
^a^ ≥50% reduction in MADRS total score from baseline
^b^ MADRS total score ≤10
Data are not available for all patients at all time points
 *p* values are for paired *t* test between groups, with statistically significant differences shown in bold
